# Editorial: The Changing Panorama of Diabetes Outcomes: Novel Complications and Novelties in Classical Complication

**DOI:** 10.3389/fendo.2021.816481

**Published:** 2022-01-13

**Authors:** Khalid Siddiqui, Ernesto Maddaloni

**Affiliations:** ^1^ Strategic Centre for Diabetes Research, College of Medicine, King Saud University, Riyadh, Saudi Arabia; ^2^ Department of Experimental Medicine, Sapienza University of Rome, Rome, Italy

**Keywords:** diabetes, complications, special issue, heart failure, Alzheimer’s disease, retinopathy

The global exponential rise in diabetes incidence is undoubtedly one of the most demanding health concerns. As it predicts a substantial increase in the number of chronic complications, with profound effects on the overall burden of metabolic disorders accompanied by a worsening of the quality of life, an increase in the demand on health services and an increase in the health-related economic costs.

Complications such as cardiovascular disease, nephropathy, retinopathy and diabetic neuropathies represent the “classical” and most studied diabetes-related long-term complications. The endothelium appears to be the common substrate hit by hyperglycaemia, insulin resistance and the other cardiovascular risk factors. The discovery of novel disease pathways in this regard is contributing to the identification of potential therapeutic targets, which promise to offer thrilling opportunities for tackling vascular complications of diabetes (1). An aggressive therapy against classical cardiometabolic risk factors (2) is leading to decreasing rates of cardiovascular mortality (3). However, the increased cardiovascular risk specifically conferred by diabetes is intact, causing a higher rate of mortality for this group (4). Furthermore, novel clinical implications of classical vascular complications have been recognized. In particular, while atherothrombosis has always been considered the main cardiovascular complication of diabetes (5), both epidemiological and clinical data showed that heart failure develops at increased rates and with worse outcomes in individuals with diabetes. Indeed, it is now well established that pathways leading to cardiovascular disease in patients with diabetes go far beyond atherosclerotic coronary artery disease and also include microvascular dysfunction, finally leading to impaired myocytes contraction and relaxation. As a matter of fact, heart failure is a driving cause of cardiovascular morbidity in people with diabetes, claiming for novel research clarifying the mechanisms underlying bidirectional link between heart failure and diabetes, in order to ameliorate cardiovascular outcomes among people with diabetes (6).

Furthermore, the systemic effects of diabetes are extensive, and novel complications of diabetes have been recently recognized, since strong epidemiological association between diabetes and other high-prevalence disorders such as osteoporosis and neurological diseases have been found (7, 8). Undeniably, the increase in both diabetes incidence and life expectancy has led to an overall increase in the years of life spent with diabetes, a chronic and debilitating condition. This is leading to a progressive aging of the global population with diabetes, with an increased prevalence of age-related disorders. However, uncertainties exist about the relationship between aging and diabetes, with diabetes apparently accelerating the aging of organs and tissues and the onset and progression of age-related disorders.

In this respect, a link between diabetes, vascular dysfunction, and dementia is gaining increasing importance, as highlighted in several articles of this special article collection.

Indeed, this Research Topic has broadly discussed the different novel complications and novelties in classical complications. Berlanga-Acosta et al. explained in an updated review article the role of insulin resistance and impaired cerebral glucose metabolism in Alzheimer’s disease (AD) and other neurodegenerative processes. AD is primarily described as a metabolic disorder in which amyloid accumulation may appear as a by-product of more proximal events, especially in the late-onset form. The bridge between AD and type 2 diabetes, activation of c-Jun N-terminal kinase (JNK) pathway with the ensued serine phosphorylation of the insulin response substrate (IRS)-1/2 may be at the crossroads of insulin resistance and its subsequent dysmetabolic consequences, as discussed in the manuscript.

Pan et al. instead reviewed recently published cardiovascular outcome trials (CVOTs) of three new classes of anti-diabetic agents, namely dipeptidyl peptidase-4 inhibitors (DPP-4is), glucagon-like-peptide 1 receptor agonists (GLP-1RAs), and sodium glucose cotransporter-2 inhibitors (SGLT-2is) or SGLT-2 and SGLT-1 dual inhibitors, with respect to their effect on heart failure.

In classical microvascular complication of diabetes, diabetic retinopathy (DR) is a significant cause of vision loss and a research subject that is constantly being explored for new mechanisms of damage and potential therapeutic options. Rao et al. delineates mechanisms of damage secondary to triglyceride and cholesterol dysmetabolism vs. mechanisms secondary to diabetes to add clarity to the pathogenesis behind each proposed mechanism, in order to elucidate the synergistic, additive, and common pathways of damage in diabetic retinopathy. Continuing with this, Augustine et al. explain lipids can undergo modification as a result of interaction with reactive oxygen species (ROS), supporting a role for lipid peroxidation and ALE formation in the onset and development of this condition. Potential therapeutic approaches to prevent lipid peroxidation and lipoxidation reactions in the diabetic retina are also considered, including the use of antioxidants, lipid aldehyde scavenging agents and pharmacological and gene therapy approaches for boosting endogenous aldehyde detoxification systems. Abdelrahim et al. summarizes available scientific evidence on the occurrence of hypoglycaemic events (HE) and the effects of different moderators on the incidence of HE among patients with T2DM during the holy month of Ramadan. Hypoglycaemia is an acute complication associated with diabetes management, increasing cardiovascular disease risk.

In this Research Topic an original research paper by Liu et al. investigated how the pathological hyperglycaemia and hyperinsulinemia seen in the prediabetic stage may regulate the expression of microRNA-21 (miR-21) and affect the downstream insulin signalling pathways, leading to endothelial cell dysfunction and early renal damage.

Finally, Ke et al. highlights the role of histone deacetylase in the pathogenies of diabetic cardiomyopathy, and Jannapureddy et al. provide insights into exploring potential preventative and therapeutic strategies regarding the roles for Aldose Reductase (AR) in the causes and consequences of diabetic cardiovascular disease and the status of AR Inhibitors in Clinical Trials.

## Author Contributions

The editorial article has been written and reviewed by EM and KS they co-edited the Research Topic on *The Changing Panorama of Diabetes Outcomes: Novel Complications and Novelties in Classical Complication*. All authors contributed to the article and approved the submitted version.

## Conflict of Interest

The authors declare that the research was conducted in the absence of any commercial or financial relationships that could be construed as a potential conflict of interest.

## Publisher’s Note

All claims expressed in this article are solely those of the authors and do not necessarily represent those of their affiliated organizations, or those of the publisher, the editors and the reviewers. Any product that may be evaluated in this article, or claim that may be made by its manufacturer, is not guaranteed or endorsed by the publisher.
